# A dermatology E-learning programme is perceived as a valuable learning tool in postgraduate general practice training

**DOI:** 10.5116/ijme.612f.3d6c

**Published:** 2021-09-29

**Authors:** Michelle J. H. Verheijden, Herm Martens, Sylvia Heeneman

**Affiliations:** 1 Department of Family Medicine, Care and Public Health Research Institute, Maastricht University, Maastricht, the Netherlands.; 2 Department of Dermatology, Maastricht University Medical Centre+, Maastricht, the Netherlands; 3 Department of Pathology, School of Health Professions Education, Maastricht University, the Netherlands

**Keywords:** E-learning, dermatology, mixed-methods, postgraduate education, family medicine

## Abstract

**Objectives:**

This study aims to explore GP residents' knowledge retention and residents' and clinical teachers' perception on the learning value of a dermatology E-learning programme.

**Methods:**

The study used a mixed-method design with a convergent parallel collection of data. GP residents (n=21) were selected through purposive sampling and were randomized to an E-learning group (n=12) or a traditional teaching methods group (n=9). The pre-and post-intervention knowledge tests of the E-learning group were compared using paired-samples t-tests. Post-knowledge tests scores of both groups were compared using independent t-tests. Cronbach's coefficient α was used to calculate the internal consistency of the questions used in the knowledge tests. Individual semi-structured interviews and clinical teachers (n=16) were conducted and analyzed using King's template analysis.

**Results:**

The E-learning group showed a significant increase in mean knowledge test scores from 58.92% (SD=9.55%) to 64.92% (SD=13.65%) (t_(11)_=2.258, p=0.045, Cohen’s d=0.51). The pre-knowledge test consisted of 46 items (Alpha=0.78), and the post-knowledge tests consisted of 45 items (Alpha=0.90). Interview data showed that the E-learning programme aided GP trainees' learning process and favoured a mixed-method teaching design, in which E-learning is used in parallel to the traditional teaching methods.

**Conclusions:**

A dermatology E-learning programme appeared an effective strategy in resident's knowledge acquiring. The key users' perceptions, both residents and clinical teachers, indicated that E-learning was feasible and helpful for learning processes. Further research is required to evaluate the implementation of E-learning programmes in parallel to regular teaching programs.

## Introduction

In medical schools and residency training programs, dermatology training is limited, leading to both knowledge gaps in dermatological pathology as well as low confidence in the performance of skin examinations and management of dermatological conditions.^1^ Previous studies have shown that the dermatological diagnostic ability of General Practitioners (GPs) is suboptimal.^2^^-^^4^ Cutaneous disorders form a significant percentage of the GPs workload (15% of the GP consultations a day).^5^^,^^6^ Consequently, adequate diagnosis and treatment of dermatological conditions by GPs is essential to optimize patient referrals to dermatologists, prevent misdiagnoses and their impact on patient health, as well as to increase trust and satisfaction among patients in the competency of their GPs.^4^^,^^6^

Therefore, it is important to improve the dermatological background and experiences of future GPs by providing appropriate dermatological training during their residency.^2^^,^^7^ However, resident shifts and work-hour restrictions typically interfere with daily teaching or lecturing.^8^ Also, the ongoing changing context of medical education demands a more active, self-steering attitude from students over time.^9^ Thus, other formats of teaching, like E-learning programmes, should be explored to establish effective learning.

E-learning or online learning is defined as 'any educational intervention mediated electronically via the Internet'.^10^ A growing body of literature recognizes the importance of E-learning in medical education.^11^^-^^15^ In comparison to traditional teaching methods (lectures, teacher-led discussions, and group work assignments), E-learning methods use a format that is available and comparable for all users.^16^^,^^17^

Recent studies that have evaluated the effect of E-learning formats in dermatology have shown that students valued its visual and interactive aspects.^7^^,^^14^^,^^18^^-^^22^ Thereby, an E-learning programme in combination with traditional teaching methods resulted in improved retention of knowledge regarding dermatological topics.^19^ Moreover, Fransen and colleagues reported a positive effect of E-learning programmes on acquiring dermatology knowledge of undergraduate medical students. Students appreciated the visual images, multiple-choice questions and feedback on the answers, which facilitated the recognition of dermatological conditions.^7^

Nonetheless, there are limited insights into the effect of E-learning in workplace-based postgraduate education. As such, less is known about the determinants and frequency of E-learning utilization in postgraduate medical education.^8^ The aforementioned lack of dermatological knowledge, the variable working shifts, the different learning context (students versus residents), and fewer insights on residents' learning effect indicates a need to better understand the GP residents' perceptions of a dermatology E-learning and how it affects their learning processes.

Furthermore, there is little known about clinical teachers' perceptions on embedding E-learning programmes in the educational programme.^23^^-^^25^ Students or postgraduates and clinical teachers are educational partners, and their relationship determines the better understanding of contents, opportunities to learn with peers and the interaction within the group. Therefore, it is required to achieve a better understanding on how teachers respond to E-learning programmes and on their acceptance.^24^

The present study aims to determine GP residents' perceptions of the learning effect of a dermatology E-learning programme. Furthermore, we aim to determine the clinical teachers' perceptions on embedding and using the E-learning programmes in the traditional teaching methods for GP residents. The following research questions were studied: (1) what is the effect of a dermatology E-learning programme on the acquisition of GP residents' dermatological knowledge? (2) what are GP residents' perceptions on the learning effect of a dermatology E-learning programme? and (3) what are clinical teachers' perceptions on embedding and use of an E-learning programme in dermatology in the traditional teaching methods for GP residents?

## Methods

### Design, setting and participants

**Figure 1:**
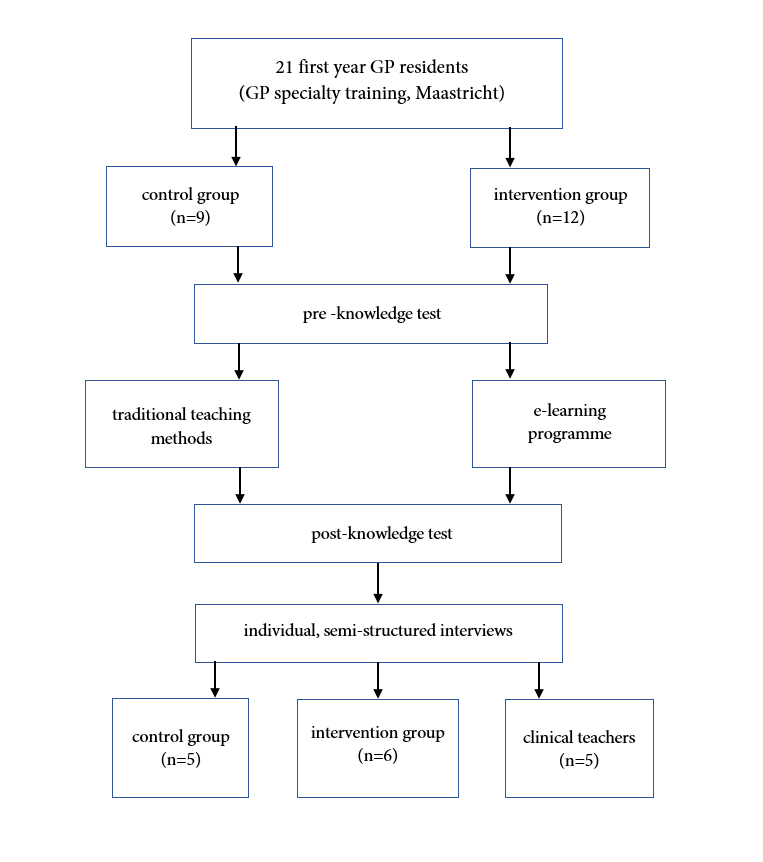
Study design and flowchart of study participants

The study took place in the period from May 2019-August 2019 and used a mixed-method design (Figure 1) with a convergent parallel collection of data in order to create a synergistic understanding, including qualitative data (individual semi-structured interviews) and quantitative data (results of pre-and post-intervention knowledge tests.)^7^^,^^26^

Participants were first-year GP residents and clinical teachers at the GP Specialty Training programme of Maastricht University, the Netherlands. The residency programme consists of three years, in which residents participate in weekly education days organized by the GP Specialty Training Programme. The content of these days includes lectures, case-based lectures and group work about different fields of medicine.

GP residents (n=21) from the spring 2019 cohort were asked to participate in the study in the first educational meeting. After consent, residents (n=21) were randomized into an intervention group and a control group: (1) GP residents who were not participating in the traditional teaching methods but did have access to and were participating in the E-learning programme (n=12) and (2) GP residents who were participating in the traditional teaching methods but did not have access to and were not participating in the E-learning programme (n=9). For the interviews, eleven GP residents gave consent, six residents of the E-learning programme group and five residents of the traditional teaching group. The traditional teaching methods consisted of two scheduled educations sessions (180 minutes) addressing dermatological topics provided by clinical teachers from the GP Specialty Training.

The online dermatology E-learning programme, Education in Dermatology (ED), is developed by dermatologists and is easily accessible from any desktop computer, laptop, and smartphone with an internet connection. The programme consisted of 31 clinical cases about cutaneous problems. The cases contained images and multiple-choice questions regarding descriptions, diagnosis and management of cutaneous problems. Answers and feedback were provided with examples of important visual features necessary to evaluate skin disorders. In addition, web-based links to learning materials were provided within the E-learning programme.

Clinical teachers (n=5) spending more than 6 hours per week teaching were approached via e-mail or in person. Four teachers with access to the E-learning programme and one teacher with no access to the E-learning programme participated in the interviews. The Ethical Review Board (ERB) of the Netherlands Associations for Medical Education (NVMO) approved the procedures of this study.

Figure 1. Study design and flowchart of study participants

The figure provides information on the study design and study participants (GP residents). Twenty-one first year GP residents were divided into two groups (control group and intervention group). After two knowledge tests, semi-structured interviews were conducted with GP residents' and clinical teachers' to explore perception about the E-learning programme.

In the decision-making procedures, the ERB applies guidelines based on ethical principles from existing frameworks and codes of conduct (e.g., the Declaration of Helsinki, last revised in 2013). Participating trainees and clinical teachers gave written informed consent. All data were anonymized with codes.

### Data collection

#### Quantitative data

In order to identify the effect of the E-learning programme on knowledge acquisition, the residents completed a pre-and post-knowledge test, i.e., before and after participating in the traditional teaching method or the E-learning programme.

Dermatologists of Maastricht University Medical Centre+ (MUMC+) developed the pre-and post-knowledge tests. Each test contained 45 multiple-choice questions regarding diagnosis, management and treatment of common dermatological conditions. The tests mainly focused on different levels of learning: knowledge, application and thinking/problem-solving ability. The questions are part of an existing validated question bank used for summative assessment during clerkships at MUMC+. To ensure validity and reliability, all questions were critically reviewed by a dermatologist and a course instructor (HM, SM) before using these in the pre-and post- knowledge tests. Moreover, internal consistency was investigated by calculating Cronbach's alpha.

#### Qualitative data

Semi-structured individual interviews of approximately 60 minutes with GP residents and clinical teachers were conducted by the first researcher (MV) after the post-knowledge test took place. The interview guides (Appendix A and Appendix B) contained open-ended questions probing for expectations, perceptions, personal experiences and learning activities by using the E-learning programme or traditional teaching method.

The interviews were audio-recorded, transcribed verbatim and analyzed using template analysis.^27^ The audio recordings were deleted after the transcription process. The results will be presented through summaries and quotes.

### Data analysis

#### Quantitative data

All data are expressed as means with corresponding standard deviation (SD) unless indicated otherwise. The pre-and post-intervention knowledge tests of the intervention group were compared using paired-samples t-tests. Post-knowledge tests scores of the intervention and the control group were compared using independent t-tests. Statistical significance was set at p<0.05. Effect sizes (Cohen's d) with corresponding 95% confidence intervals were calculated for the quantitative comparison between the two groups. Cronbach's coefficient α was used to calculate the internal consistency of the questions used in the knowledge tests. A Cronbach's alpha between ≥0.70 and ≤0.95 was classified as good.^28^ All analyses were performed using the Statistical Package for Social Sciences (SPSS version 24).

#### Qualitative data

The analysis of the transcripts was independently done by MV and a second researcher (SH) using template analysis.^27^ Template analysis were performed using Atlas.ti software (version 8.0). The interviews continued until thematic saturation was reached. The thematic saturation was determined by the research team following these criteria: (1) if new data could be fitted in categories that were already devised, (2) if no new insights, themes, issues or counter-example/cases arose, and (3) consensus within the research team was reached about the notion of saturation with the collected and analyzed data.^27^

Analysis of interviews 1-5 with the GP residents of the intervention group was labelled, coded by MV, and crosschecked by SH. The outcomes were compared, and differences were discussed until consensus was reached, which resulted in an initial template used in interviews 6-9 (four residents of the E-learning programme group and one resident of the control group). As coding proceeded, constant comparison defined the characteristics of each category and resulted in an adapted initial template, which was used for the interviews with the clinical teachers. Finally, by examining and re-examining the data from the intervention, the control group, as well as the clinical teachers' group, the relationships among the major categories were explored, and no new insights were obtained. At this point, thematic saturation was reached.

## Results

### Quantitative data

In total, 21 GP residents were included, and all subjects (9 residents of the control group and 12 residents of the intervention group) completed the six-week E-learning programme or the two education sessions as part of the traditional teaching methods. No drop-out was seen.

For the pre-knowledge test, no statistical analysis could be performed because of missing data in the control group due to technical problems with the E-learning programme (data was not saved). The pre-knowledge test consisted of 46 items (Alpha=0.78), and the post-knowledge tests consisted of 45 items (Alpha=0.90). The intervention group showed a significant increase in knowledge test scores from the pre- (M=58.92%, SD=9.55%) to the post-knowledge test (M=64.92%, SD=13.65%, t_(__11)_= 2.258, p = 0.045, Cohen’s d = 0.51), suggesting that the E-learning intervention moderately benefitted the knowledge acquisition of GP residents. There was no significant difference in post-knowledge test scores of the control (M=66.38%, SD=15.78%) and intervention group (M=64.92%, SD=13.65%, t_(__18)_=0.351, p=0.730, Cohen’s d=0.10).

### Qualitative data

In the following paragraphs, we will explore the different primary themes and provide clarifying quotes.

### Format

The content provided by the E-learning programme was considered to be uniform and set a basic level for everyone. GP residents indicated to be easily overwhelmed by the many textbooks that are available for traditional education and often did not know where to start. The E-learning programme provided a starting point for their learning.

“It [E-learning programme] is a good way to acquire knowledge. I find it less trouble than random opening a book or an NHG-standard [Dutch protocols designed especially for GPs], and not knowing where to start. The knowledge you acquire from books or NHG-standards does not lasts and at a certain moment, you have read it all.” -  GP resident (intervention group - interview 6)

Residents participating in the control group indicated a lack of uniformity in the selected clinical cases and had the perception that the learning effect of the weekly education sessions were mainly determined by the quality of the teacher, the quality of the group, and/or the quality of the selected cases. However, the collaborative approach, mainly the dialogue and discussion regarding clinical cases, was positively perceived and lead to retention of main messages. Moreover, involving GP residents in real-world clinical cases and linking new information to prior knowledge required effective communication and collaboration among clinical teachers, GP residents, and others. GP residents from the intervention group did not miss this collaborative approach. Nonetheless, it was stated that the dialogue and discussion within the traditional teaching method (in other education sessions they attended) was appreciated. However, it was also noted that the interactivity of these education sessions strongly depended on the skills of the clinical teacher.

“The group discussion stimulates you actively to work and think about the problem. Not only listening, also actively taking part in the discussion, instead of passive listening, for me, that is the same as passively reading a book. The important thing is that you become activated. Thereby, you are being forced to think for yourself and be able to explain your thoughts to the group. Eventually, you can easier understand why you are giving that specific answer to the group.” - GP resident (control group - interview 5)

Clinical teachers suggested to establish a clearer structure/framework for the E-learning programme to allow better understanding of all the different dermatological conditions (e.g., by classifying groups of dermatological conditions), instead of the offered more fragmented dermatological cases. Therefore, GP residents could possibly miss links between theory (learning about dermatological conditions) and practice (recognizing and treating a variety of dermatological conditions in clinical practice). During the education sessions, clinical teachers described that these links between theory and practice would be made easier via dialogue and discussion and thereby help GP residents on embedding dermatological knowledge.

A few barriers of the E-learning programme were related to technical issues, e.g., the slowness' of the programme.

### Agency

GP residents used the E-learning programme autonomously and in their own phase and time (e.g., during clinic hours when a patient dropped out, during a free afternoon, or at home). They indicated that autonomous learning by using the E-learning programme enabled them to find more additional information about the cases by using the offered links and references from the E-learning programme. The references and the links helped the GP residents to review wrong answers. Moreover, they could freely choose to use the links for additional information, and they became acquainted with other materials. Therefore, they not only studied and learned independently, they also became more self-managing and easily used the links to the websites to find out more and study the mechanisms of disease.

“The fact that you can use it [E-learning programme] in between two patients; if a patient drops out and you have more time left. Furthermore, I can just open and use the E-learning programme for a couple of minutes in between work.” – GP resident (intervention group - interview 1)

The E-learning programme push-notes maintained the regular use of the E-learning programme and offered instant stimulation to the GP residents to learn. They appreciated this stimulation, as the amount of time they must spend at the clinical workplace is substantial with little time left for actual study, and therefore to schedule time for study was easily forgotten.

GP residents in the control group indicated that recall of the acquired dermatological knowledge was not easy given due to the limited teaching hours and no stimulation to revisit teaching material. Thereby, little time was left during the traditional teaching methods for GP residents to individually acquire more information about specific dermatological conditions, which was not discussed during the education sessions. Furthermore, there was no direct-follow up possible and no time for GP residents to study and learn in their own phase and time.

“In a group [traditional teaching methods], you cannot find more information about a topic you forgot about, for example: Which cream? Which steroid class? That is not something you are going to look up for yourselves in an education session. However, if you work on an E-learning programme at home, you are able to immediately choose yourselves to find more information about it. Thereby, you can determine for yourselves on what topic you have to find more information, and thereby you can easier adjust it to yourselves and to your knowledge.” – GP resident (control group - interview 5)

### Exposure to cases

The high-resolution images in the E-learning programme allowed the GP residents to gain a deeper understanding of the range of clinical presentations and provide more exposure to dermatological conditions. In addition, GP residents were able to identify their own knowledge gaps (e.g., different kinds of therapies for dermatological conditions). GP residents valued learning through clinical cases, which they also recognized in clinical practice. In addition, they appreciated focusing on a selection of dermatological topics instead of being overwhelmed by many comprehensive textbooks (related to theme **'Format'**).

GP residents from the intervention group as well as the control group experienced a lack of basic dermatological knowledge and preferred more exposure to dermatological education.

“I am lacking a bit of knowledge, knowledge concerning dermatological conditions that are often seen in general practice.” – GP resident (control group - interview 5)

The selected cases from the E-learning programme contained common dermatological conditions, rare dermatological conditions, and life-threatening dermatological conditions. Clinical teachers indicated that the selected cases and the associated multiple-choice questions from the E-learning programme were encountered in the daily clinical practice, and therefore enabled the GP residents to acquaint a good balance in dermatological knowledge for various items of conditions.

“My opinion about the content [E-learning programme] was that it did not consist of any rare conditions. The cases [dermatological conditions] are commonly encountered in the general practice. Those are relevant cases that you will actually see in general practice.” – Clinical teacher (access E-learning programme - interview 4)

In contrast to the selected cases from the education sessions, the GP residents themselves determined the input of the cases. Therefore, it is possible that a rare, or life-threatening, or even a common dermatological condition could be missed.

### Link with practice

GP residents indicated that exposure to dermatological cases in practice was a valuable learning experience. The recognition of clinical cases from the E-learning programme in practice was perceived as helpful, offered repetition, and confirmed their dermatological knowledge. It also enabled the use of that specific case to optimize their consultation and to consolidate their knowledge.

“I am an active student, so I have to see something, I have to do something, and from that experience, I learn something, thus, this [E-learning programme] offers me a perfect solution. I would rather see it than that I have to take a book and read it. Thus, I prefer the situation [E-learning programme]; seeing things, checking, getting feedback, and more, practicing and recognizing.” – GP resident (intervention group - interview 2)

GP residents in the control group elaborated on prior experiences with other E-learning programmes and prefer that E-learning programmes would provide authentically clinical cases related to daily clinical practice. This link between theory and practice was present during GP residents' education sessions and was valued. In these education sessions, GP residents met in a group and worked on several cases of a patient with a dermatological condition. The GP residents themselves have chosen these clinical cases from their own practice. However, in a number of selected cases, the diagnosis was not certain. GP residents felt uncertain and insecure about the possibility of missing diagnoses. Clinical teachers noted that especially the first year GP residents were looking for certainties, and missing information in the education session or in practice can be led to insecurity about their clinical eye.

“It is common in the group first year GP residents are very eager not to miss any lessons, not all of them, but it is a repeating theme that plays a role by all of them, by some more than the others, but it still remains a repeating theme that comes back in the first year and which is often mentioned.” – Clinical teacher (access E-learning programme - interview 5)

Within the E-learning programme, this insecurity was not present as feedback was provided via the programme to the GP residents. GP residents noted that by receiving feedback from the E-learning programme led to a deeper understanding of the different dermatological conditions.

Clinical teachers stated that instantly triggering GP residents with questions and clinical cases combined with a self-chosen time and medium fits the GP resident of today perfectly. Thereby, linking the digital learning environment of the E-learning programme to the traditional teaching methods.

“I think, to my opinion, that it [E-learning programme] fits the current GP resident perfectly. From my own experience, I see more often GP residents, who appreciate it when learning is interactive, in the format of a quiz, something they can actively participate to, as long as they are getting entertained. I think they value that the most, and to my opinion, I got the idea, that, the more serious learning like spending hours learning from a book, is, how do I have to put it, is something, that through the years has become less sexy.” – Clinical teacher (access E-learning programme - interview 4)

## Discussion

The aim of this study was to determine first-year GP residents' and clinical teachers' perceptions and the learning effect in GP residents of a dermatology E-learning programme versus traditional teaching methods. Therefore, we conducted a study that combines a quantitative and qualitative design.

The quantitative data showed a significant learning effect through the E-learning programme in the intervention group. Due to the missing data of the pre-knowledge test in the control group, it was not possible to determine whether the learning effect of the E-learning programme differed from the traditional teaching methods. The post-knowledge test scores' showed little difference between the intervention- and the control group. Fransen and colleagues used the same validated question bank for the knowledge tests as this study, and therefore it is possible that the knowledge tests could not have connected well to the prior knowledge of the GP residents.^7^ Postgraduates, for instance, GP residents, have more experience in clinical practice than undergraduate medical students and have more existing (basic) dermatological knowledge due to more clinical experience. A number of participants in the interviews also expressed the lack of alignment of the tests.

The qualitative data explored the learning mechanisms of GP residents. Four primary themes were identified via template analysis: format, agency, exposure to cases, and link with practice.^27^ Overall, GP residents valued learning through authentically clinical cases, which allow them to link theory to practice. GP residents indicated that the E-learning programme had a number of advantages, such as the uniform format, the accessibility, and incentive for regular use. On the other hand, GP residents receiving traditional teaching methods appreciated the dialogue and group discussion that enabled interaction and link of theory to practice. However, GP residents following traditional teaching methods stated that they could not acquire dermatological knowledge in their own phase and time, were not able to recall certain clinical cases, and wished for more exposure to dermatological conditions. Clinical teachers stated that these links between theory and practice would be easier to achieve through dialogue and discussion. Moreover, they indicated that the E-learning programme fits current GP residents perfectly because it enables linking the digital learning environment of the E-learning programme to the traditional teaching methods.

Our results corroborate the ideas and findings in literature.^14^^,^^18^^,^^19^^,^^21^ Silva and colleagues analyzed and evaluated the impact of a dermatology E-learning programme on students' learning.^19^ The E-learning programme combined with the traditional course (blended learning) significantly increased students' knowledge about dermatology, compared to students who solely received traditional teaching methods. Therefore, Silva and colleagues concluded that the use of an E-learning programme, in combination with traditional teaching methods, improved retention of dermatological knowledge.^19^ The qualitative data of this study also explored GP residents' learning mechanisms, and we found that all GP residents valued to learn through authentically clinical cases by linking theory to practice: the E-learning programme by providing a wide selection of clinical cases followed by links to websites and the traditional teaching methods by providing clinical cases that were selected by the GP residents themselves from their own clinical experience. By incorporating E-learning programmes in the residency training programme, GP residents benefit from the advantages of both methods.

In accordance, Campbell and colleagues demonstrated that the use of virtual learning environments was associated with higher assignment marks than students who participated in face-to-face discussions.^29^ In the current study, the test scores of the intervention group improved significantly.

Although the effect size was relatively small and non-significant between the post-knowledge test scores (control group versus intervention group), the qualitative data analysis suggested that the E-learning programme can be used as a meaningful learning activity, in addition to any teaching method. Thereby, methods can benefit from each other, and the E-learning programme will not repeat subjects of the traditional teaching methods used in the setting of this study but provides a deeper understanding of acquired dermatological knowledge.

Some studies have failed to show a difference in learning effects between E-learning programmes and traditional teaching methods.^30^^-^^32^ However, despite the lack of a significant difference in test results, students preferred the online learning module format to the traditional teaching method format. The online learning module took less time, and a clearer structure was provided.^33^ The interview data in this study also pointed out that GP residents appreciated the more uniform format, the constant availability of the teaching material, and the equal content for everyone.

### Limitations

The findings of this study have to be seen in the light of some limitations. Firstly, the missing data of the pre-knowledge test of the control group made it impossible to determine whether the learning effect of the E-learning programme differed from the traditional teaching methods. Secondly, the E-learning programme was only evaluated in one context and setting (Maastricht University). Thirdly, participation was voluntary. Thereby, the possibility exists that motivated GP residents and clinical teachers participated in this study, however of the spring 2019 cohort, all residents participated. Fourthly, the relatively small sample size of the control and intervention groups during quantitative data collection.

Given the average effect of the e-learning intervention on knowledge acquisition in undergraduate medical education,^7^ the power calculation suggested a sample size of 11 GP residents per group (22 GP residents in total). In the time allowed, we could only recruit 21 GP residents in total, so we are aware that our study is underpowered. Therefore, our results may not be generalizable to other areas and medical curricula. Despite the sample size, clear themes emerged from the qualitative data collection that is consistent with prior literature. Thus, this would suggest that the findings are of value for medical educators.

### Implications for research and/or practice

GP residency programs could benefit from integrating E-learning technologies in their traditional teaching methods. Thereby, a link between theory and practice was enabled and eventually led to a higher level of dermatological knowledge and improved the dermatological diagnostic ability of GP's. The acquired insights could help to design effective E-learning programmes in which (digital) learning is supported for students as well as clinical teachers. For example, E-learning programmes are tailored to traditional teaching methods in which clinical teachers give GP residents guidance and structure by systematically describing dermatological conditions, and E-learning programmes provide instant stimulation via authentically clinical cases from practice. Thereby, giving GP residents structure to their clinical practice and eventually facilitate them to salve and understand dermatological conditions.

## Conclusions

The aim of the present study was to explore GP resident's knowledge retention and resident's and clinical teachers' perception of the learning value of a dermatology E-learning programme. This study showed that the use of an E-learning programme in dermatology for GP residents was perceived as a valuable learning tool. The E-learning programme resulted in an improvement in GP residents' dermatology knowledge. In addition, GP residents and clinical teachers perceived that the E-learning programme enabled GP residents to acquire dermatological knowledge in their own phase and time, to link theory to practice, and to recall clinical cases.

Given the advantages of both teaching methods, E-learning programmes and traditional teaching methods should be combined to be of benefit for each other. Future studies should evaluate and focus on the perceptions of learners and teachers to enable a fit-for-purpose implementation of E-learning programmes in traditional teaching methods.

### Acknowledgements

The authors wish to thank all GP residents and clinical teachers who participated in this study.

### Conflicts of Interest

The authors declare that they have no conflict of interest.

## Supplementary material

Supplementary file 1 Appendix A. Interview guides - GP-residents (Maastricht University, the Netherlands, 2019)

Supplementary file 2 Appendix B. Interview guides - GP-residents (Maastricht University, the Netherlands, 2019)
