# Supplementary material for: A dermatology E-learning programme is perceived as a valuable learning tool in postgraduate general practice training
Source: Int J Med Educ. 2021 Sep 29;12:169–78. doi: 10.5116/ijme.612f.3d6c (PMC8994645; doi:10.5116/ijme.612f.3d6c)
Supplement: Supplementary file 1 — Appendix A. Interview guides - GP-residents (Maastricht University, the Netherlands, 2019) [file ijme-12-169-S1.pdf]

Appendix A

Interview guides - GP-residents (Maastricht University, the Netherlands, 2019)

Interview guide A: perceptions of first year GP residents on their learning processes (E-learning programme)

Expectations in relation to E-learning methods

- Did the E-learning programme cover the content you expected? Why or why not?
- Did the E-learning programme covered the knowledge gaps you missed in your dermatological knowledge?
- Did the content of the E-learning programme cover your expectation?
- What did you learn from the E-learning programme?

Structure, usability and content of E-learning application

- How relevant is the content of the E-learning programme to cases in daily practice/clinical encounter?
- How relevant are the cases of the E-learning programme, for you as a learner?
- Did you use the web links to external websites that were provided in the E-learning programme to find more background information? If so, how did you use the web links? If not, what was the reason for not using it?
- Which part of the E-learning programme did you experience as the most useful and interesting?

Time

- What was the (mean) amount of time you spend on this E-learning programme?
- What was the (mean) amount of time you spend for studying (after completion of the cases provided by the E-learning programme)?

Interactivity

- What are your thoughts on the interactivity provided by the E-learning programme?

Overall experience

- Can you identify three concepts or ideas you have learned in this E-learning programme?

Interview guide B: perceptions of first year GP residents on their learning processes (traditional teaching methods)

Expectations in relation to the traditional teaching methods

- Did the traditional teaching methods cover the content you expected? Why or why not?
- Did the traditional teaching methods covered the knowledge gaps you missed in your dermatological knowledge?
- Did the content of the traditional teaching methods cover your expectation?
- What did you learn from the traditional teaching methods?

Structure and content of the traditional teaching methods

- How relevant is the content of the traditional teaching methods to cases in daily practice/clinical encounter?
- Did you miss anything in the traditional teaching methods?
- Which part of the traditional teaching methods did you experience as the most useful and interesting?

Time

- What was the (mean) amount of time you spend for studying (after attending the traditional teaching methods)?

Interactivity

- What are your thoughts on the interactivity provided by the traditional teaching methods?

Overall experience

- Can you identify three concepts or ideas you have learned in from the traditional teaching methods?
