# Supplementary material for: A dermatology E-learning programme is perceived as a valuable learning tool in postgraduate general practice training
Source: Int J Med Educ. 2021 Sep 29;12:169–78. doi: 10.5116/ijme.612f.3d6c (PMC8994645; doi:10.5116/ijme.612f.3d6c)
Supplement: Supplementary file 2 — Appendix B. Interview guides - GP-residents (Maastricht University, the Netherlands, 2019) [file ijme-12-169-S2.pdf]

Appendix B

Interview guides - GP-residents (Maastricht University, the Netherlands, 2019)

Interview guide C: perceptions of clinical teachers (access to the E-learning programme) on embedding and using E-learning programmes in the traditional teaching methods for GP residents

Expectations in relation to E-learning

- What were your expectations regarding the E-learning programme?
- Do you think that in the future the E-learning programme can replace the traditional teaching methods?
- Do you expect that GP residents eventually score higher on dermatological topics by the use of the E-learning programme?
- Do you think the E-learning programme has an added value?

Structure, usability and content of E-learning application

- How relevant is the content of the E-learning programme to cases in daily practice/clinical encounter?
- Should you also recommend using E-learning for other domains?

Overall experience

- Can you identify three concepts or ideas you have learned in this E-learning programme?

Interview guide D: perceptions of clinical teachers (no access to the E-learning programme) on embedding and using E-learning programmes in the traditional teaching methods for GP residents

Expectations in relation to E-learning

- What are your expectations in general regarding the use of E-learning programmes?
- Do you think that in the future E-learning programmes can replace the traditional teaching methods?
- Do you expect that GP residents eventually score higher on dermatological topics by the use of E-learning programmes? Do you think E-learning programmes have an added value?

Structure, usability and content of E-learning application

- What content would you like to incorporate in E-learning programmes?
- Should you also recommend using E-learning for other domains?

Overall experience

- Can you identify three concepts or ideas you would like to learn in an E-learning programme?
